# An Analysis of Suicide Risk Factors among Farmers in the Midwestern United States

**DOI:** 10.3390/ijerph18073563

**Published:** 2021-03-30

**Authors:** Andrea Bjornestad, Courtney Cuthbertson, Jessie Hendricks

**Affiliations:** 1 Department of Counseling and Human Development, South Dakota State University, Wenona Hall 302, Box 0507, Brookings, SD 57007, USA; 2 Department of Human Development and Family Studies, University of Illinois, 904 W. Nevada Ave., Urbana, IL 61801, USA; cuthbert@illinois.edu; 3 Department of Mathematics and Statistics, South Dakota State University, Architecture, Math & Engineering Building 209, Box 2220, Brookings, SD 57007, USA; Jessie.Hendricks@jacks.sdstate.edu

**Keywords:** farmer, rancher, mental health, suicide, anxiety, depression, self-blame coping, social support, farm stress

## Abstract

Research on the complex relationships of variables contributing to farmer suicide is limited. The purpose of the study was to examine factors associated with suicide risk through the use of standardized instruments measuring psychological (depression, anxiety), social (social support), and contextual factors. A questionnaire was completed by 600 farmers in the Midwestern United States. A multiple linear regression model was used to analyze associations with suicide risk (SBQ-R), including depression (PHQ-9), anxiety (GAD-7), Brief COPE subscales (BC), social support (MSPSS), and select demographic and farming characteristics. The only variable that emerged as having a significant relationship with the natural log-transformed suicide risk score was coping through self-blame. While suicidality is often considered the outcome of mental illness, our findings do not suggest that suicide risk among farmers is related to mental illness, and a further examination of self-blame as a coping strategy is warranted.

## 1. Introduction

From 1999 to 2018, suicide rates increased 35% in the United States, resulting in a lower life expectancy for Americans [1,2,3]. The trend in suicide is not distributed evenly across the population; middle-age White men die by suicide more frequently than other groups [4], and suicide rates in 2018 were higher in most rural areas than urban ones [2]. Attempts to further understand this phenomenon have included analyzing suicide rates by occupation, finding that people who work within agriculture (producers) have suicide rates that are much higher than the general population [5]. With 3.4 million producers in the U.S. [6], this is an important population to understand and support through public health programs and interventions. Agricultural producers include individuals who regularly utilize land for the production of crops or commercial livestock, and the majority of farmers in the U.S. are white men with an average age of 58 [7]. Data related to producers is inconsistent and limited, and while risk factors for producer suicide are widely discussed in popular media, research studies are scant. In this study, we investigate connections between depression, anxiety, and suicide risk along with demographic and farming characteristics, farming-specific stressors, coping strategies, and social support among producers in four Midwestern U.S. states. Identifying characteristics associated with depression, anxiety, and suicide risk among producers can help to develop public health programs and strategies to effectively intervene and support producers.

Over the past twenty years, only approximately 30 articles have explored depression and suicide risk among primary agricultural producers in the U.S., with over half of those publications based on three research studies [8]. The available evidence about agricultural producers’ mental health generally demonstrates that producers have higher psychological distress, depression, and anxiety than the general population. Prevalence rates of depression in producers have varied from 7.4% to 24% [9] with risk factors for depression including legal problems, income loss, health declines, losing something of sentimental value [10] and low social support from friends and family [11]. Depressed mood has also been associated with having an additional off-farm job, stress, previous injury, pesticide exposure [9], and financial stress [8]. 

Even less is known about anxiety and suicide risk in U.S. producers. Most studies have occurred internationally with prevalence rates of anxiety among producers ranging from 19% to 71% [12,13]. Various factors have been suggested to contribute to anxiety in producers including climate change with uncertain weather conditions [14], market prices, government regulations, and relationship issues [15]. Specific farmer characteristics such as education and financial knowledge were also related to increased anxiety [16]. 

With the heightened concern regarding the declining mental health of producers, it is imperative to begin to explore the complex relationships of psychological, social, and contextual factors in relation to suicide. Depression has a strong association with suicidal ideation and attempts; however, it is a weak predictor of suicide [17] suggesting suicide risk is more than mental illness. Researchers in Australia have attempted to extract the underlying contextual variables contributing to suicide in farmers via qualitative methods. When exploring environmental factors related to producer suicide, Perceval et al. [18] identified eight subthemes: extreme climatic events, isolation, service availability, access to and frequent use of firearms, death and suffering of animals, government legislation, technology, and property values. Results of other qualitative studies have suggested distressed relationships and ineffective coping mechanisms may be contributors to farmer suicide. Kunde et al. [19] used a psychological autopsy method by interviewing close significant others of male farmers who died by suicide. Six interrelated themes were identified: masculinity (obeying masculine social norms), uncertainty and lack of control in farming, feelings of failure in relationships and farming, escalating mental and physical health problems, maladaptive coping, and access to lethal means. All of these themes suggest that mental illness may not be the primary contributor to farmer suicide. 

Research on the complex relationships of variables contributing to farmer suicide is extremely limited not only due to the low number of research studies in the U.S., but also due to several methodological issues. First, in larger data sets such as those used by the CDC, farmers are often categorized with forestry and fishing, and their role within farming is not clarified. Additionally, suicide data are limited in how occupation is identified; often, farmers may be listed as “self-employed,” rather than as farmers. This paper makes unique contributions to the literature through the use of standardized instruments measuring psychological (depression, anxiety, coping behaviors, suicide risk), social (social support), and contextual factors, making findings more comparable with those among the general population.

## 2. Materials and Methods

To address the low amount of information available about mental health and suicide risk among farmers and to investigate factors associated with suicide risk, we surveyed agricultural producers in the Midwest United States. A list of 4000 producers who either farmed at least 1000 acres or who owned a dairy farm was purchased from US Farm Data. A hard-copy survey was mailed to all 4000 producers (1000 per state) from Kansas, Michigan, Missouri, and South Dakota. The survey included demographic questions, the Suicide Behaviors Questionnaire-Revised (SBQ-R) [20]), the Patient Health Questionnaire-9 (PHQ-9) [21], the General Anxiety Disorder 7-item scale (GAD-7) [22], the Brief COPE questionnaire [23], and the Multidimensional Scale of Perceived Social Support (MSPSS) [24]. 

### 2.1. Participants

A total of 600 responses were received (25% Kansas, 33% Michigan, 13% Missouri, 29% South Dakota) with a response rate of 15%. Survey respondents were, on average, 63 years of age (M = 63, SD = 12.5), with the youngest at 20 years and the oldest at 94 years. The majority of participants were male (81% male, 19% female), married (83% married, 17% not married), and not currently or previously in the military (82% no military service, 18% military service). Approximately half of participants had received a college degree or higher (51% college degree or higher, 49% less than college degree). A majority of respondents indicated that they were primary decision-makers as the principle or primary owner/operator of the farm (64%), 30% indicated that they were partner owners/operators of the farm and participate in shared decision making, and 7% indicated that they were extended family of the principle owner/operator. Only 22% of participants were first generation farmers, while more established generations composed the remaining 78% of participants. 

### 2.2. Survey Instruments

The SBQ-R [20] is a self-report measure composed of four items with each item assessing a different aspect of suicidality. Item 1 inquires about lifetime suicide ideation and attempt, while item 2 asks about the frequency of suicidal ideation over the past 12 months. Item 3 evaluates the threat of suicidal behavior, and item 4 assesses self-reported likelihood of suicidal behavior. The SBQ-R is calculated as one score using all four items, with a range of 3 to 18. The threshold for significant risk of suicide using the SBQ-R in the general population is a score of 7 or higher. The SBQ-R has empirical support for use as a risk measure of suicide in both nonclinical and clinical samples [20]. 

The PHQ-9 [21] is a 9-item self-report measure that is widely used in health care settings to screen for depression in the general population [25,26]. The items in the PHQ-9 are calculated into a score with a range from 0 to 27. Using the PHQ-9, the cutoff for mild depression is a score of 5–9, moderate depression is 10–14, moderately severe depression is 15–19, and severe depression is 20 or more. The PHQ-9 was found to be without gender bias [27] and is effective across diverse racial and ethnic groups [28]. 

To measure anxiety, we use the GAD 7-item scale (GAD-7) [29]. Scores for each GAD-7 item are totaled, and total scores range from 0 to 21. Using the GAD-7, the cutoff for mild anxiety is 5–9, moderate anxiety is 10–14, and severe anxiety is 15–21. The GAD-7 is a valid and efficient assessment for screening for Generalized Anxiety Disorder in clinical settings and research, [21] and the scale has been used in other studies of agricultural producers [12,13].

The Brief COPE is a 28-item self-report measure that gauges effective and ineffective coping behaviors. The measure is often used in healthcare settings and can assist in determining a person’s primary coping style. Rather than calculating a total score, the Brief COPE is calculated into 14 scales, each derived from two of the Brief COPE items: self-distraction, active coping, denial, substance use, emotional support, use of informational support, behavioral disengagement, venting, positive reframing, planning, humor, self-acceptance, and self-blame [23]. Scores for each sub-scale range from 2 (low use) to 8 (high use). The measure has been validated with diverse racial and ethnic populations [30,31].

The MSPSS [24] is a 12-item self-report measure that examines perceived social support on three subscales: family, friends, and a significant other. Each item is scored on a 7-point scale, ranging from 1 (very strongly disagree) to 7 (very strongly agree) [24]. Each subscale is the average of four items, and subscale scores range from 1 (low support) to 7 (high support). The MSPSS has strong factorial validity confirming the three subscales and is a widely used measure with strong factorial validity and internal consistency reliability across varying groups [32,33,34]. Descriptive statistics for the scored SBQ-R, PHQ-9, GAD-7, Brief COPE (14 subscales), and MSPSS (3 subscales) are presented in Table 1 and Table 2.

### 2.3. Data Analysis

A multiple linear regression model was used to investigate the relationship between independent variables of mental health, social support, coping techniques, and demographic variables, and one dependent variable, suicide risk (SBQ-R). Mental health variables included in the model were scores for anxiety (GAD-7) and depression (PHQ-9). Social support variables were calculated categories from the MSPSS into support from a significant other, support from family, and support from friends. Coping techniques were calculated from the Brief COPE. Demographic variables included in the model were age, gender, marital status, education level, and military experience. Farming demographic variables were included as well, particularly farming generation and farm role related to decision-making. 

A natural log transformation of the SBQ-R was necessary to better satisfy several of the assumptions for a multiple linear regression model (i.e., normally distributed error terms with equal variance). Prior to fitting the model, high correlations were noted among several of the independent variables, which had potential for multicollinearity in the model. After fitting the model, several diagnostics were performed (such as checking variance inflation factors) to ensure there were no major effects on the model due to multicollinearity. The diagnostics confirmed that the effects were minimal, so no further action was required and results could be interpreted. Responses were included only if participants had answered all of the questions used to score the GAD-7, PHQ-9, and SBQ-R, and the demographic questions. A total of 344 complete responses were used to fit the model. All analyses were performed in R [35].

## 3. Results

Within our sample, the mean score of anxiety symptoms was 3.3 (SD 4.5), where 27% (*n* = 148) met the criteria for generalized anxiety disorder. Related to depression using the PHQ-9, the mean score was 3.8 (SD 4.7). While 29.3% (*n* = 159 of 539 complete responses) met the criteria for major depression, over three-quarters (75.4%) reported symptoms of depression. As the ninth item on the PHQ-9 is about suicide risk, we also analyzed the sample without the ninth item. Using the PHQ-8, the mean was 3.7 (SD 4.5), where 28.7% (*n* = 162 of 565 complete responses) met the criteria for depression. Regarding suicide risk score, we found that the sample had a mean score of 3.8 (SD 1.7), with 7.6% of the sample (*n* = 41) at significant risk of suicide.

The results of the multiple linear regression model appear in Table 3. The multiple R-squared for the multiple linear regression model was 0.2973 and the adjusted R-squared was 0.2372. The R-squared value can be interpreted that 29.73% of the variation in the natural log-transformed suicide risk score (SBQ-R) can be explained by the model. We used a Benjamini and Hochberg adjustment for multiple testing to control the false discovery rate at 5%. Adjusted p-values are given in the furthest right column of Table 3 and can be compared to α= 0.05. At this level of significance, two independent variables emerge as having a significant linear relationship with the natural log-transformed suicide risk score (SBQ-R): depression symptom experience (PHQ-9) and self-blame as a coping strategy. A one-unit increase in PHQ-9 scores is associated with an expected increase of 0.023 units of the natural log-transformed SBQ-R, holding all other variables fixed. In other words, a one-unit increase in depression symptoms increases the untransformed suicide risk score (SBQ-R) by 2.3% (exp(0.023) = 1.023). A one-unit increase in self-blame is associated with an expected increase of 0.06 units of the natural log-transformed SBQ-R, holding all other variables fixed. That is to say that a one-unit increase in self-blame is associated with an expected increase in the untransformed suicide risk score of 6.2% (exp(0.060) = 1.062). No significant results were observed for the remaining independent variables, including anxiety symptoms, other forms of coping, various types of social support, demographic variables, or farming-related variables.

One potential issue with Model 1 is that the PHQ-9 includes a question asking the individual whether they had thoughts they would be better off dead or of harming themselves in some way (question 9), which is assessing for suicide risk. The PHQ instrument is also valid with the first 8 items of the PHQ-9, so we tested the model using the PHQ-8 to ensure the significance found in Model 1 was not due to the inclusion of the ninth PHQ item about suicide risk. 

We analyzed the survey responses using the same multiple linear regression technique, using the natural log transformation of the SBQ-R to better satisfy several assumptions for the model. The model was fitted using a total of 347 complete observations. The multiple R-squared for the model was 0.288 and the adjusted R-squared was 0.2278. This can be interpreted to state that 28.8% of the variation in the natural log-transformed SBQ-R can be explained by the model. Again, we used the Benjamini and Hochberg adjustment for multiple testing and the adjusted p-values are given in the furthest right column of Table 4. 

In Model 2, we found that only one independent variable emerged as having a significant linear relationship with the natural log-transformed suicide risk score: coping through self-blame. The adjusted p-value for self-blame was 0.006, and we found that a one-unit increase in self-blame was associated with an expected increase of 0.065 units of the natural log-transformed SBQ-R. That is, a one-unit increase in self-blame is associated with an expected increase of 6.7% (exp(0.065 = 1.067) in the untransformed suicide risk score, holding all other conditions constant. Removing the ninth PHQ item assessing suicide risk in this model showed that experiencing depressive symptoms was no longer significant. 

## 4. Discussion

Chronic stress has been associated with mental illness including depression and anxiety [36,37]. In this study, 29.3% of producers met the criteria for depression. Although the PHQ-9 was not utilized for diagnostic purposes, the results suggest a higher prevalence among farmers than those reported in previous studies [9]. This increase in prevalence rate has occurred during a time of shifting market conditions and trade policies, extreme weather changes, and distressed finances with the frequency of calls to farm aid hotlines resembling the 1980s farm crisis [38]. 

In addition to the increased concern regarding depressive symptoms among producers, very little is known about the prevalence of anxiety in this population. In this sample, 27% of producers met the criteria for generalized anxiety disorder on the GAD-7, which is higher than the estimated 19.1% in the general population [39]. However, our prevalence rate was lower than the 71% found in young farmers [13] and 58% of Canadian farmers experiencing varying levels of anxiety [40]. Although studies have explored the sources of anxiety for producers, very few have examined the human characteristics contributing to anxiety [16] and how those characteristics may impact suicide risk.

Individuals with poor mental health or mental illness may perceive surveys differently. Those with poor mental health or mental illness are more likely to be suspicious of research studies, experience difficulties understanding survey questions, and may skip questions or fail to return the survey [41]. Thus, it is possible that the prevalence rates of depression and anxiety in the current study are underreported. 

The current study examined the complex relationships of psychological, social, and contextual variables in relation to suicide risk scores among producers. An initial model including demographic variables and scores from the PHQ-9, GAD-7, Brief COPE, and MSPSS were analyzed which showed depressive symptom experience on the PHQ-9 and self-blame as a coping strategy reaching significance. However, one item on the PHQ-9 assessed suicide risk; therefore, it was determined that a model should be tested without the item. Only coping with self-blame reached significance when the PHQ-8 was utilized. Other studies have also found significant associations between self-blame and suicide risk, although such studies were not among farming populations [42,43].

The explanations for increased suicides among producers have been complex. Suicides have occurred in the absence of higher rates of mental illness in farming communities suggesting that multiple factors may contribute to suicide risk [44]. The emergence of self-blame as the risk factor for suicide among producers in the current study suggests that suicide risk in agriculture may be related to attributions. Self-blame as a coping mechanism may be adaptive or maladaptive. Janoff-Bulman [45] described two types of self-blame: behavioral and characterological. Behavioral self-blame pertains to the belief that one’s misplaced behavior can be modified and the consequence corrected; thus, self-blame is within one’s control and is adaptive. Those that engage in behavioral self-blame concentrate on the specific behavior and may attempt to repair situations that result in failures. Characterological self-blame involves self-critical, maladaptive methods of blaming one’s character for failures. Essentially, characterological self-blame influences self-esteem and is viewed as unchangeable by the individual resulting in shame. 

Farmers who engage in behavioral self-blame may be more apt to focus on the future and determine how to avoid the repetition of a negative outcome. This coping strategy is adaptive as the goal would be to prepare for positive future outcomes. Conversely, farmers who engage in characterological self-blame believe they are deserving of the negative outcome. This coping strategy is maladaptive as they do not focus on the future; rather, they are stuck in the deservingness of the negative outcome from the past and attribute the failure as a flaw in their character.

Kunde et al. [19] identified two distinct suicidal process pathways in farmers: acute situational (e.g., romantic relationship issues, financial concerns) and protracted (long-term psychiatric disorder). Characterological self-blame may be a potential explanation for the acute situational pathway proposed by Kunde et al. [19]. It is possible that farmers that are high risk for suicide internalize their difficulties (e.g., relationships, financial distress) as a defect in their character and feel as if they cannot effect change. They cognitively attack their self-esteem and dismiss the possibility that the situation can be repaired leading to a downward spiral of hopelessness and helplessness. The maladaptive self-criticism and lack of perceived control may be factors in the development and progression of suicidal thoughts and ideations even in acute situations.

The items on the Brief COPE that explore self-blame include: “I’ve been criticizing myself”, and “I’ve been blaming myself for things that happened.” Both items have negative connotations with the first item hinting at a current self-evaluation, and the second item describing an evaluation of something that happened in the past. Considering the items do not specifically measure behavioral or characterological self-blame, future research may involve the modification of items to clearly make a distinction. 

Additionally, noteworthy in the current analysis are some of the non-significant findings. We found no significant relationship in the multiple linear regression model between any of the demographic characteristics or social support and suicide risk as we would have expected based on findings from the broader literature. For instance, in the general population, women are more likely to attempt suicide and men are more likely to die by suicide [4,46]. Among farming populations, suicide is more common among men [47]. Military veterans have a higher risk of suicide [48]. Education level is generally protective against suicide among the general population [49,50]. For the age groups with the highest suicide rates, there was a social belief that a high school diploma was sufficient for economic opportunities as an adult [50]. Whereas education may have been protective among the general population during economic downturns, education is not protective within-occupation when assessing risk and rates among agricultural producers. Further, agriculture is a debt-driven industry where producers face financial uncertainty due to fluctuating commodity prices leading to an inability to precisely predict financial outcomes far in advance. It is possible that the types of challenges in agriculture and the stresses created by them overcome any protective effect of particular demographic characteristics among farmers compared to the general population.

We also did not find any significant relationship between social support and suicide risk, regardless of whether social support comes from a significant other, family, or friends. This may be due to the tendency of farming to be within families rather than with other occupations where a work role is occupied by one individual with little to no involvement from their network of personal friends and family. In other words, the potentially large overlap between personal and work networks for agricultural producers may result in producers feeling they are less able to get support from their personal networks due to family involvement in work.

This study is unique as standardized instruments were utilized to measure psychological (depression, anxiety, coping behaviors, suicide risk), social (social support), and contextual factors, making findings more comparable with those among the general population. The limitations to the current study include having a non-random sample, as well as stigma related to mental health that may have led to social desirability bias in how producers responded to the questions. Agriculture is a diverse industry with the various commodities produced; as such, our response rates by commodity were not high enough to analyze for between-group differences based on commodity. This is an area for future research as anecdotal evidence from Wisconsin, Iowa, and Michigan indicates that dairy farmers may experience higher rates of suicide and suicide attempts than those producing other commodities [51,52]. Our data are also limited as we sampled amongst farmers with at least 1000 acres of land, which eliminated smaller-scale farmers as well as farmworkers from the sample.

The response rate for the survey was 15%. Although 15% is a low response rate for mailed questionnaires, it is a typical response rate for surveys mailed to agricultural producers. Mailed survey response rates with farmers in recent studies have ranged from 15% [53] to 28% [54]. Survey response rates in rural areas have greatly declined due to factors such as lack of trust with researchers, numerous surveys received, chaotic farm schedules, and age [53]. Producers may be more likely to complete the survey if they feel the benefits outweigh the costs, and the survey is relevant to them [55]. Additionally, length of survey may have been a factor as crop producers have limited time to complete a survey with the best months being January and February. The current study included five scales, and the majority of producers will only complete surveys that take less than ten minutes to finish [53]. Our surveys were mailed between the months of April through November, and thus did not reach producers during slower months.

## 5. Conclusions

The findings of our study that producers have higher rates of depression, anxiety, and suicide risk, in combination with the existing literature, demonstrate that this population is in need of public health supports for mental wellbeing. Public health programs could focus on mental health literacy and stress management strategies to reduce self-blame among producers. Considering self-blame was the only variable that had a significant association with suicide risk, the results demonstrate continued need for further investigation of factors related to suicide risk among agricultural producers, specifically the types of self-blame; potential future studies could analyze the relationship of agriculture-specific stressors with suicide risk. Additional studies are needed to develop specific strategies for suicide prevention among agricultural producers, and to better understand factors that would support their well-being.

## Figures and Tables

**Table 1:** Percent and *n* of respondents by calculated levels of PHQ-9 (depression), GAD-7 (anxiety), and SBQ-R (suicide risk).

Measure	Level	Percent	*n*
PHQ-9	None	24.68	134
	Minimal	46.04	250
	Mild	18.42	100
	Moderate	6.81	37
	Moderately severe	2.03	11
	Severe	2.03	11
GAD-7	None/minimal	73.04	401
	Mild	17.85	98
	Moderate	4.92	27
	Severe	4.19	23
SBQ-R	Not at significant risk	92.41	499
	At significant risk	7.59	41

**Table 2:** Mean and standard deviation for PHQ-9, GAD-7, SBQ-R, Brief COPE, and MSPSS.

Measure	Variable	Mean	SD
	PHQ-9	3.792	4.703
	GAD-7	3.301	4.479
	SBQ-R	3.796	1.731
Brief COPE	Active coping	3.969	1.812
	Behavioral disengagement	2.348	0.827
	Denial	2.306	0.839
	Emotional support	3.254	1.514
	Instrumental support	3.113	1.399
	Positive reframing	3.800	1.615
	Self-distraction	3.442	1.506
	Substance use	2.351	0.934
	Venting	3.033	1.267
	Acceptance	4.253	1.870
	Humor	2.994	1.293
	Planning	4.153	1.922
	Religion	4.259	2.074
MSPSS	Significant other	5.596	1.526
	Friends	5.053	1.352
	Family	5.478	1.329

**Table 3:** Model 1: Multiple linear regression model to analyze associations with the natural log-transformed suicide risk (SBQ-R), including depression (PHQ-9), anxiety (GAD-7), Brief COPE subscales (BC), social support (MSPSS), and select demographic and farming characteristics.

Variable	Estimate (B)	exp(B)	Std. Error	t-value	Pr(>|t|)	adj. p
(Intercept)	1.209	3.350	0.172	7.034	0.000	--
PHQ9	0.023	1.023	0.007	3.323	0.001	0.013
GAD7	−0.010	0.990	0.007	−1.479	0.140	0.421
BC—Self distraction	0.012	1.012	0.017	0.744	0.457	0.650
BC—Active coping	−0.016	0.984	0.017	−0.984	0.326	0.582
BC—Denial	−0.044	0.957	0.025	−1.787	0.075	0.273
BC—Substance use	0.012	1.012	0.019	0.627	0.531	0.710
BC—Emotional support	0.043	1.044	0.018	2.404	0.017	0.113
BC—Instrumental support	−0.019	0.981	0.019	−0.973	0.331	0.582
BC—Behavioral disengagement	−0.012	0.988	0.028	−0.442	0.659	0.773
BC—Venting	0.040	1.041	0.019	2.042	0.042	0.209
BC—Positive reframing	0.000	1.000	0.017	0.004	0.997	0.997
BC—Planning	−0.014	0.986	0.017	−0.805	0.422	0.632
BC—Humor	−0.006	0.994	0.016	−0.398	0.691	0.778
BC—Acceptance	0.008	1.008	0.013	0.595	0.552	0.710
BC—Religion	−0.015	0.985	0.011	−1.390	0.166	0.440
BC—Self blame	0.060	1.062	0.017	3.468	0.001	0.013
MSPSS—Sig. other	−0.009	0.991	0.020	−0.463	0.644	0.773
MSPSS—Family	0.031	1.031	0.024	1.277	0.203	0.440
MSPSS—Friends	−0.037	0.964	0.018	−2.000	0.046	0.209
Age	−0.001	0.999	0.002	−0.824	0.410	0.632
Gender—Male	0.126	1.134	0.050	2.512	0.013	0.113
Marital status—not married	0.044	1.045	0.047	0.946	0.345	0.582
Military—yes	0.086	1.090	0.049	1.752	0.081	0.273
Education—College degree or higher	−0.001	0.999	0.034	−0.020	0.984	0.997
Farm Role—Partner owner/operator (shared decision-making)	−0.102	0.903	0.081	−1.251	0.212	0.440
Farm Role—Principle/primary owner/operator (primary decision-maker)	−0.111	0.895	0.083	−1.335	0.183	0.440
Farm Generation—Greater than first generation	−0.002	0.998	0.040	−0.051	0.959	0.997

**Table 4:** Model 2: Multiple linear regression model to analyze associations with suicide risk, removing the 9th item of the PHQ-9.

Variable	Estimate (B)	exp(B)	Std. Error	t-value	Pr(>|t|)	adj. p
(Intercept)	1.194	3.300	0.171	6.975	0.000	--
PHQ8	0.019	1.019	0.007	2.668	0.008	0.095
GAD7	−0.008	0.992	0.007	−1.246	0.214	0.444
BC—Self distraction	0.012	1.012	0.017	0.740	0.460	0.653
BC—Active coping	−0.016	0.984	0.017	−0.946	0.345	0.517
BC—Denial	−0.042	0.959	0.025	−1.678	0.094	0.318
BC—Substance use	0.011	1.011	0.019	0.567	0.571	0.734
BC—Emotional support	0.045	1.046	0.018	2.477	0.014	0.095
BC—Instrumental support	−0.019	0.981	0.019	−0.995	0.320	0.517
BC—Behavioral disengagement	−0.007	0.993	0.028	−0.255	0.799	0.899
BC—Venting	0.044	1.045	0.019	2.267	0.024	0.130
BC—Positive reframing	−0.001	0.999	0.017	−0.050	0.960	0.989
BC—Planning	−0.017	0.983	0.017	−0.982	0.327	0.517
BC—Humor	−0.006	0.994	0.016	−0.382	0.703	0.825
BC—Acceptance	0.009	1.009	0.013	0.648	0.518	0.699
BC—Religion	−0.014	0.986	0.011	−1.344	0.180	0.444
BC—Self blame	0.065	1.067	0.017	3.756	0.000	0.006
MSPSS—Sig. other	−0.008	0.992	0.020	−0.421	0.674	0.825
MSPSS—Family	0.031	1.031	0.024	1.250	0.212	0.444
MSPSS—Friends	−0.036	0.965	0.018	−1.966	0.050	0.226
Age	−0.002	0.998	0.002	−0.985	0.325	0.517
Gender—Male	0.124	1.132	0.050	2.467	0.014	0.095
Marital status—not married	0.046	1.047	0.047	0.974	0.331	0.517
Military—yes	0.092	1.096	0.049	1.874	0.062	0.238
Education—College degree or higher	0.000	1.000	0.034	−0.013	0.989	0.989
Farm Role—Partner owner/operator (shared decision-making)	−0.103	0.902	0.080	−1.294	0.197	0.444
Farm Role—Principle/primary owner/operator (primary decision-maker)	−0.115	0.891	0.082	−1.409	0.160	0.444
Farm Generation—Greater than first generation	−0.001	0.999	0.040	−0.022	0.982	0.989

## Data Availability

Data are not publicly available.
